# HIV Impairs Lung Epithelial Integrity and Enters the Epithelium to Promote Chronic Lung Inflammation

**DOI:** 10.1371/journal.pone.0149679

**Published:** 2016-03-01

**Authors:** Kieran A. Brune, Fernanda Ferreira, Pooja Mandke, Eric Chau, Neil R. Aggarwal, Franco R. D’Alessio, Allison A. Lambert, Gregory Kirk, Joel Blankson, M. Bradley Drummond, Athe M. Tsibris, Venkataramana K. Sidhaye

**Affiliations:** 1 Division of Pulmonary and Critical Care Medicine, Johns Hopkins University, Baltimore, MD, United States of America; 2 Division of Infectious Diseases, Mass General Hospital, Boston, MA, United States of America; 3 Department of Epidemiology, Johns Hopkins Bloomberg School of Public Health, Baltimore, MD, United States of America; 4 Division of Infectious Diseases, Johns Hopkins University, Baltimore, MD, United States of America; Emory University School of Medicine, UNITED STATES

## Abstract

Several clinical studies show that individuals with HIV are at an increased risk for worsened lung function and for the development of COPD, although the mechanism underlying this increased susceptibility is poorly understood. The airway epithelium, situated at the interface between the external environment and the lung parenchyma, acts as a physical and immunological barrier that secretes mucins and cytokines in response to noxious stimuli which can contribute to the pathobiology of chronic obstructive pulmonary disease (COPD). We sought to determine the effects of HIV on the lung epithelium. We grew primary normal human bronchial epithelial (NHBE) cells and primary lung epithelial cells isolated from bronchial brushings of patients to confluence and allowed them to differentiate at an air- liquid interface (ALI) to assess the effects of HIV on the lung epithelium. We assessed changes in monolayer permeability as well as the expression of E-cadherin and inflammatory modulators to determine the effect of HIV on the lung epithelium. We measured E-cadherin protein abundance in patients with HIV compared to normal controls. Cell associated HIV RNA and DNA were quantified and the p24 viral antigen was measured in culture supernatant. Surprisingly, X4, not R5, tropic virus decreased expression of E-cadherin and increased monolayer permeability. While there was some transcriptional regulation of E-cadherin, there was significant increase in lysosome-mediated protein degradation in cells exposed to X4 tropic HIV. Interaction with CXCR4 and viral fusion with the epithelial cell were required to induce the epithelial changes. X4 tropic virus was able to enter the airway epithelial cells but not replicate in these cells, while R5 tropic viruses did not enter the epithelial cells. Significantly, X4 tropic HIV induced the expression of intercellular adhesion molecule-1 (ICAM-1) and activated extracellular signal-regulated kinase (ERK). We demonstrate that HIV can enter airway epithelial cells and alter their function by impairing cell-cell adhesion and increasing the expression of inflammatory mediators. These observed changes may contribute local inflammation, which can lead to lung function decline and increased susceptibility to COPD in HIV patients.

## Introduction

Combination antiretroviral therapy (ART) has transformed HIV infection from an acutely fatal disease into a chronic medical condition [1]. With longer life expectancies, emerging data indicates that individuals with HIV are more susceptible to non-infectious comorbidities such as poor lung function [2] and chronic obstructive pulmonary disease (COPD) [2–4]. After adjusting for potential confounders, individuals with HIV are 50–60% more likely to have COPD [2, 5] and individuals with poorly controlled HIV are at greatest risk for worsened spirometry, reduced diffusion capacity, and accelerated lung function decline [2, 4, 6]. HIV is also an independent risk factor for acute exacerbations of COPD [7]. Epithelial dysfunction contributes to the development of COPD and HIV alters epithelial function in multiple organs, including the lungs [8–12]. However, the cellular impact of HIV on lung epithelium is incompletely understood.

The airway epithelium acts as a physical barrier to protect the lungs from the external environment. Its ability to create this partition depends on epithelial cell-cell adhesion through intercellular junctions including the tight junctions, the adherens junctions, and desmosomes. These junctions, in particular the tight junctions and the adherens junctions, also separate the epithelial cell’s apical and basal membranes thus helping to maintain the cell’s polarity. Damage to the intercellular junctions can allow certain cytokines and growth factors to interact with their normally spatially-restricted receptors thereby increasing epithelial signal transduction to allow for repair [13, 14]. E-cadherin, a key transmembrane protein in the adherens junction, plays a critical role in modulating cell-cell adhesion as well as in initiating signaling cascades that are important in tissue remodeling and repair.

HIV strains are classified according to the co-receptor used for virus entry into the cell which is also described as virus tropism. X4 tropic viruses use the CXCR4 co-receptor for cell entry whereas R5 viruses use the CCR5 co-receptor. Dual or mixed tropic viruses either express both receptors, or have a mix of virus particles which express either receptor. Virus co-receptor usage can change throughout the course of disease [15] and studies have demonstrated that with time and in advanced stage HIV, X4 or dual tropic virus strains can be found in >50% of infected patients [16, 17]. HIV co-receptor usage can also evolve within compartments in the body. For example, studies have shown that HIV derived from sputum had a greater tendency for CXCR4 usage compared to virus derived from peripheral blood and that engagement of the CXCR4 receptor on human airway epithelial cells increased mucous production in these cells [18, 19]. We propose a mechanism by which HIV promotes the development of COPD by interacting with the lung epithelial CXCR4 receptor to alter cellular adhesive junctions and activate pro-inflammatory signaling pathways.

## Results

### HIV alters the paracellular permeability of airway epithelial cells in a dose and time dependent manner

**Fig 1:**
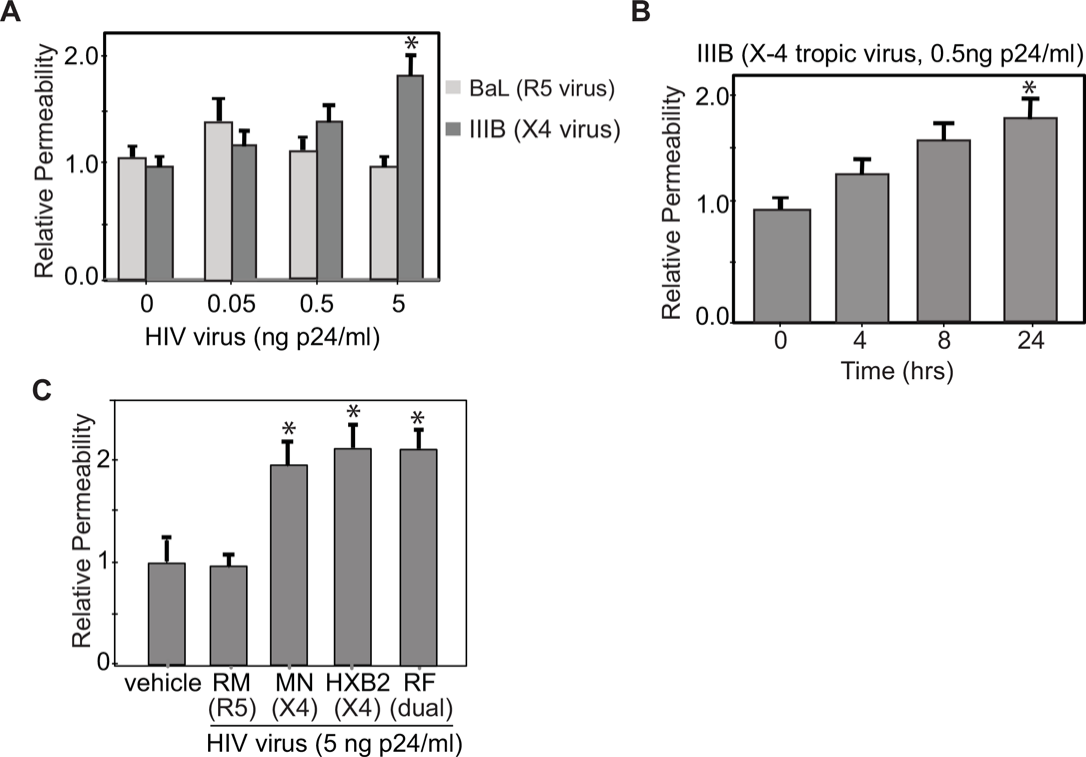
X4 tropic virus and dual tropic virus increase airway epithelial permeability. **A.** NHBE cells were exposed in the basolateral compartment to either an X4 tropic virus or an R5 tropic virus and after a four hour exposure to an X4 tropic virus, IIIB, there was a statistically significant increase in barrier permeability as measured by FITC Dextran that was not seen after four hours of exposure to an R5 tropic virus, BAL. At lower virus titers (0.05ng/ml or 0.5ng/ml), IIIB caused an increase in epithelial permeability although these changes were not statistically significant. (n = 22, *p<0.05, Anova with Bonferroni correction) **B.** Lower titers of IIIB virus (0.5ng/ml) were able to cause statistically significant increases in epithelial permeability with a 24 hour exposure. (n = 14, *p<0.05, Anova with Bonferroni correction) **C.** Both X4 tropic virus, MN and HXB2, and dual tropic virus, RF, (all at concentrations of 5ng/ml) caused similar changes in barrier permeability after four hours of exposure (n = 10, *p<0.05, Anova with Bonferroni correction).

NHBE cells were exposed in the basolateral compartment to either an X4-tropic (IIIB) or an R5-tropic (BaL) lab-adapted HIV strain and paracellular permeability was measured by a FITC Dextran assay [20, 21]. For these experiments we used the concentration of p24 in nanograms per milliliter (ng/ml) as opposed to obtaining the TCID_50_ as we believed that these pathways were triggered by virions and did not require viral replication. Specifically, cells exposed to the X4-tropic virus had increased paracellular permeability when compared to unexposed cells and cells exposed to an R5-tropic virus. These permeability changes were both dose and time dependent (**Fig 1)**. These surprising findings allowed us to use R5-tropic viruses as an ideal experimental control in subsequent experiments. During a four hour exposure to IIIB, higher virus concentrations (p24 (5ng /ml)) caused statistically significant increases in permeability however the trend toward increased barrier permeability was noted with lower concentrations of this virus (p24 (0.05ng/ml and 0.5ng/ml)) **(Fig 1A)**. There were no statistically significant changes in epithelial permeability observed in cells exposed to an R5-tropic virus (BaL) even at high virus titers. In a series of time-ranging experiments, lower titers of X4 tropic IIIB virus (p24 (0.5ng/ml)) caused a statistically significant change in barrier permeability over 24 hours **(Fig 1B)** which was not seen with R5 virus. Exposure to X4-tropic viruses (MN, HBX2) or an X4/R5 dual tropic virus (RF) at a concentration of p24 (5ng/ml) for 24 hours lead to similar increases in barrier permeability as measured by FITC Dextran assay **(Fig 1C)**. These experiments show that X4 expressing viruses were able to increase epithelial permeability in a dose and time dependent manner.

### HIV decreases E-Cadherin

**Fig 2:**
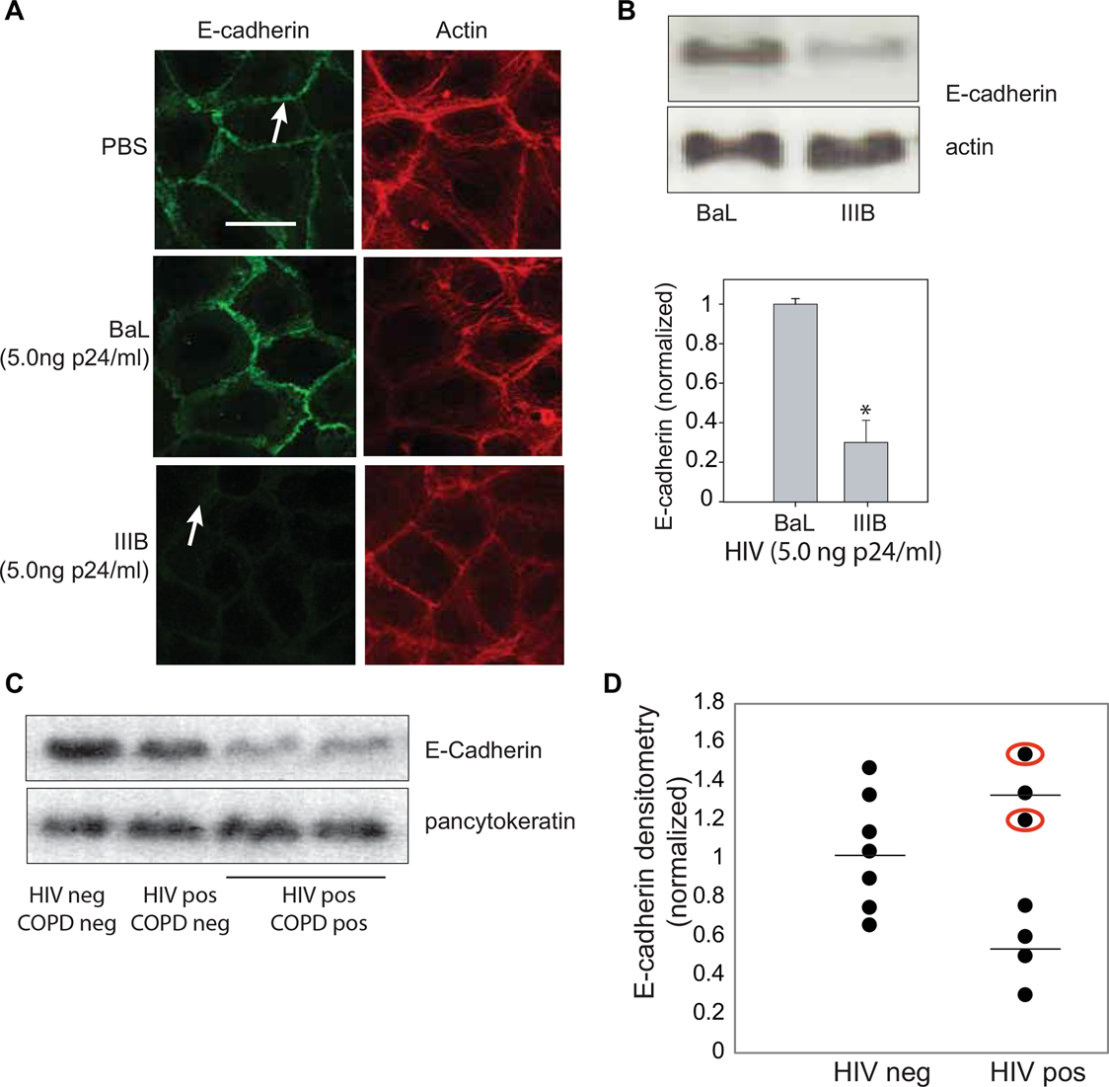
HIV reduces the abundance of E-Cadherin. **A.** Immunofluorescence shows a reduction in E-Cadherin along the basolateral membrane (white arrow) after exposure to X4 tropic virus IIIB at a concentration of p24 (5ng/ml) which was not seen after exposure to R5 tropic virus BaL at the same concentration. **B.** Western blot probing for E-Cadherin at a dilution of 1:1000 shows marked reduction in abundance of E-Cadherin following four hour basolateral exposure to X4 tropic virus but not R5 tropic virus. (n = 6 BaL and 10 IIIB samples, *p<0.05, Mann-Whitney rank sum test) **C.** By Western analysis, there is a marked reduction in E-cadherin in an epithelial cell whole cell lysate from a patient with HIV and COPD when compared to that obtained from an HIV negative COPD negative individual and an HIV positive COPD negative individual. **D.** While in the control patients, there is a normal distribution of E-cadherin level, there is a suggestion of two subpopulations of E-cadherin abundance in HIV patients. One with higher E-Cadherin levels and one with lower E-Cadherin levels. While study patients did not have viral tropism studies performed, the dots circled in red represents patients on the CCR5 inhibitor miraviroc suggesting R5 tropic disease. (Data compared using Kruskal-Wallis assessment).

In NHBE cells, E-cadherin is enriched along the basolateral surface of the cell. Reduced E-cadherin is a critical marker of poor cell-cell adhesion [22–25]. Following exposure to the X4-tropic virus, IIIB, there was decreased E-cadherin along this surface. These changes in E-cadherin localization were not observed in cells exposed to the R5-tropic virus, BaL **(Fig 2A)**. Moreover, following a four hour exposure to the X4 tropic virus IIIB at a concentration of p24 (5ng/ml), a time and dose that increased paracellular permeability, there was a reduction in total E-cadherin. Similar reduction was not seen following exposure to R5 virus **(Fig 2B)**. We confirmed that at these doses and exposure times, there was no increase in cell death following either X4 or R5 tropic virus exposure using trypan blue exclusion (not shown). Epithelial cells were obtained via bronchoscopy from a healthy HIV-uninfected control participant, an HIV-infected participant without COPD and HIV-infected participants with COPD. Those epithelial cells derived from patients with both HIV and COPD contained a much lower amount of E-cadherin as detected by immunoblotting **(Fig 2C).** To determine if HIV alone altered E-cadherin status, stored epithelial cell pellets from 7 HIV negative patients and 7 HIV positive patients (neither group had patients diagnosed with COPD) were processed to measure relative E-cadherin levels. Densitometry analysis revealed a normal distribution of E-cadherin in the HIV negative patients and a trend toward a bimodal distribution of E-cadherin levels in the HIV positive patients however; the sample size was insufficient for definitive subpopulation analysis **(Fig 2D)**. Viral tropism data was not collected for this study, but two HIV patients (circled in red) were being treated with the R5-inhibitor, miraviroc, suggesting they had documented R5 tropic disease.

**Fig 3:**
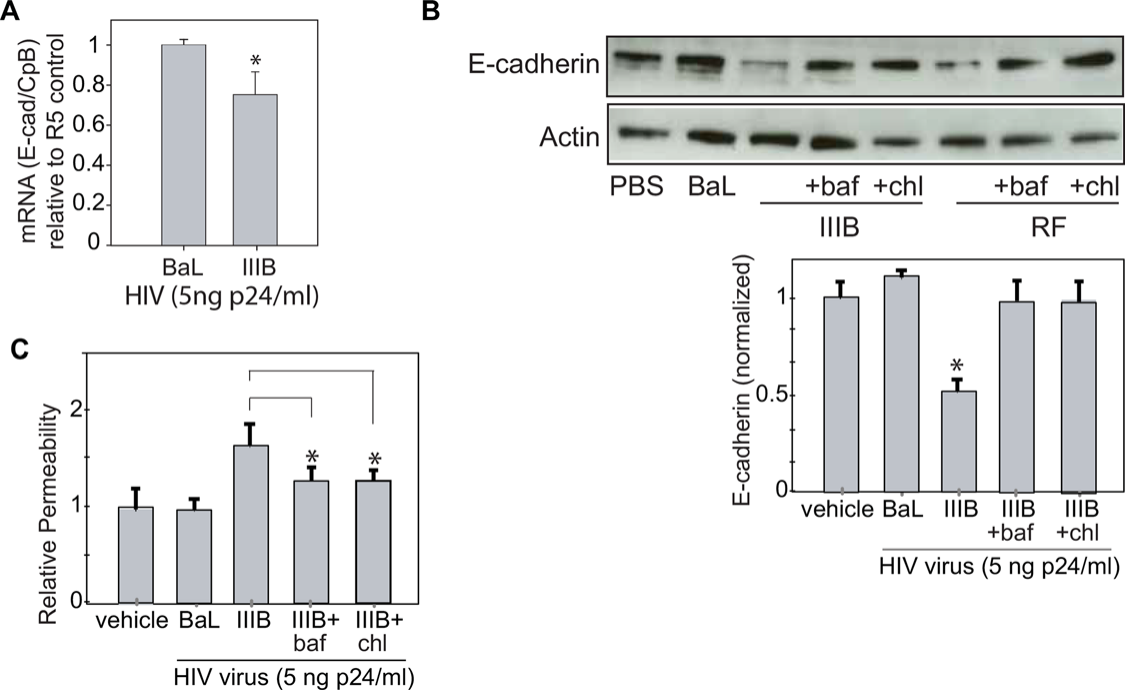
Patients with HIV have less E-cadherin in their lung epithelium. **A.** There is a slight decrease in steady state E-cadherin mRNA as detected by RT-qPCR. (n = 5, *p<0.05, student T-test) **B**. Pretreating NHBE cells for 1 hour with the lysosomal inhibitors bafilomycin or chloroquine abrogated the reduction in E-Cadherin that was seen following exposure to an X4 tropic virus (IIIB) or dual tropic virus (RF). (n = 4, *p<0.05, Anova with Bonferroni correction) **C.** Pretreating NHBE cells cells with the lysosomal inhibitors bafilomycin or chloroquine abrogated the increased permeability that was seen following exposure to the X4 tropic virus IIIB (compared to vehicle-only control which was 1:1000 DMSO in media). (n = 4, *p<0.05, Anova with Bonferroni correction).

In order to assess mechanisms leading to the decrease in E-cadherin, we assessed mRNA levels in NHBE cells exposed to the R5 virus, BaL, as compared to the X4 tropic IIIB virus. After eight hours of exposure to these viruses at a concentration of p24 (5 ng/ml), there was a small, statistically significant change in mRNA levels, although this change appeared to be less than what would be expected for that level of protein change **(Fig 3A)**. This time point was selected as it was well after the time when we first noted changes in E-cadherin in response to virus exposure. While transcriptional changes may contribute to decreased E-cadherin, these experiments did not eliminate the possibility that changes in protein degradation contributed to decreased E-cadherin. Therefore, we pretreated the cells for 1 hour with the lysosomal inhibitors bafilomycin (1μM in DMSO) or chloroquine (50μM in DMSO). Both inhibitors abrogated the changes in E-Cadherin that were seen following exposure to both an X4 virus (IIIB) and a dual tropic virus (RF) **(Fig 3B)**. As bafilomycin and chloroquine are both lysosomal inhibitors, our data indicates that X4-tropic HIV triggers E-cadherin degradation through the lysosomal pathway. In addition, pretreatment with the lysosomal inhibitors bafilomycin and chloroquine abrogated the changes in permeability that were seen following exposure to IIIB and these reductions in permeability were statistically significant **(Fig 3C)**. Collectively, these results indicate that there is a reduction in E-cadherin following exposure to both X4 and dual tropic viruses but not following exposure to R5 viruses. This reduction in protein levels occurs by both decreased transcription and lysosomal degradation of E-cadherin.

### The mechanism by which HIV disrupts the lung epithelial monolayer is CXCR4 dependent

**Fig 4:**
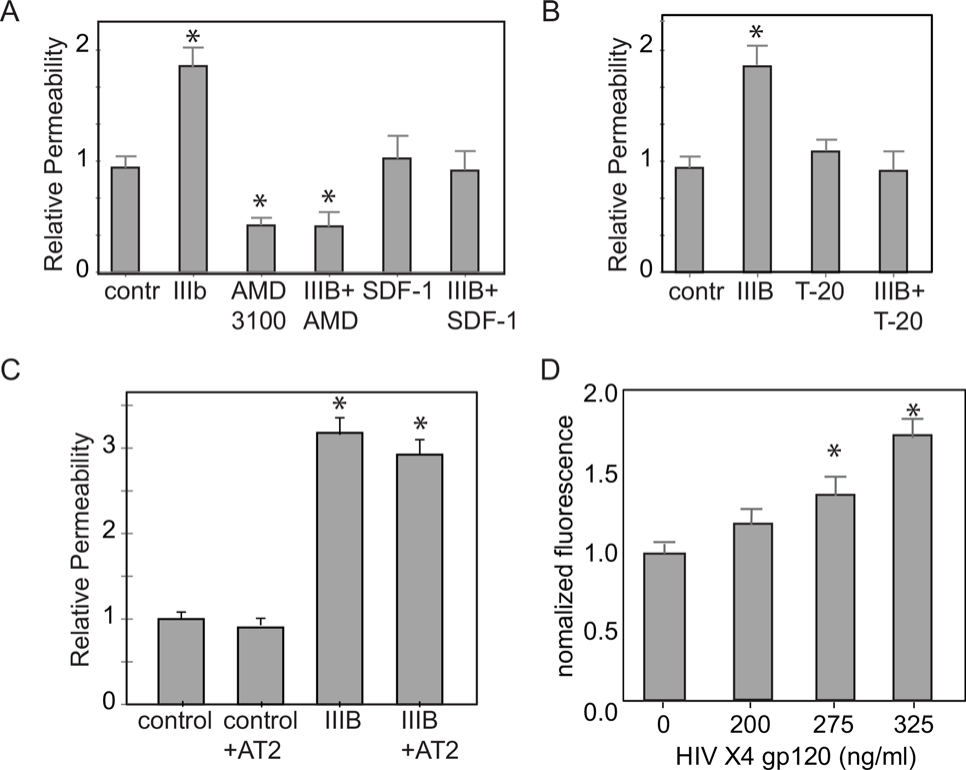
Both CXCR4 binding and viral fusion are needed to alter airway epithelial barrier permeability. **A.** Pretreating the cells with a CXCR4 antagonist, AMD3100, decreased baseline barrier permeability and prevented HIV associated barrier permeability. Pretreating the cells with CXCR4 agonist, SDF-1, prevented HIV-1 associated changes in barrier permeability. (n = 6, *p<0.05, Anova with Bonferroni correction) **B.** Pretreating the cells with the fusion inhibitor, T-20, prevented HIV associated changes in barrier permeability. (n = 8, *p<0.05, Anova with Bonferroni correction) **C.** Inactivating the X4 tropic virus IIIB with AT2, a drug that modifies the nucleocapsid but leaves the surface proteins intact, does not abrogate IIIB associated permeability changes. (n = 8, *p<0.05, Anova with Bonferroni correction) **D.** Pretreating the cells with increasing doses of recombinant X4 tropic gp120 increases epithelial monolayer permeability as determined by increased fluorescence measured in the basolateral media. Both 275ng gp120/ml and 325 ng gp120/ml caused statistically significant increases in permeability. (n = 8, *p<0.05, Anova with Bonferroni correction).

Pretreating the cells with the CXCR4 chemokine receptor antagonist AMD3100 (1μM) enhanced the baseline monolayer integrity (lower FITC permeability) and prevented HIV induced barrier disruption **(Fig 4A)** indicating that engagement with the CXCR4 receptor was necessary for barrier disruption. While treating the cells with the CXCR4 chemokine receptor agonist SDF-1 (1 μM) did not increase monolayer permeability, it did block the increased permeability associated with the IIIB virus. Cumulatively these data indicate that engagement with the CXCR4 receptor is necessary, but not sufficient, for disrupting the lung epithelial monolayer. To identify subsequent steps required to disrupt the epithelial monolayer integrity, cells were pretreated with the fusion inhibitor enfuvirtide (T-20, 1μM) [26, 27]. Enfuviritide did abrogate HIV-induced barrier disruption **(Fig 4B)**. These data suggests that both CXCR4 binding with the viral coat and viral fusion with the epithelial membrane are key steps in HIV-induced barrier disruption. As both viral binding to CXCR4 and fusion with the epithelial membrane were required, we assessed if viral entry and replication were necessary to disrupt the epithelial monolayer. Inactivating the X4 tropic virus IIIB with aldrithiol-2 (AT2, 100mM), a drug that covalently modifies the nucleocapsid while leaving the surface proteins intact [28, 29], produced similar permeability results as active IIIB virus **(Fig 4C)**. Moreover, pretreating the cells with increasing doses of recombinant X4 tropic gp120 caused statistically significant increases in monolayer permeability as was determined by increasing fluorescence measured in the basolateral media **(Fig 4D).** These data indicate that surface gp120 protein interaction and fusion are sufficient to disrupt the epithelial monolayer, however, these steps do not require a live virus and are not downstream of viral incorporation and/or replication.

### X4 but not R5 tropic virus is internalized by airway epithelial cells

**Fig 5:**
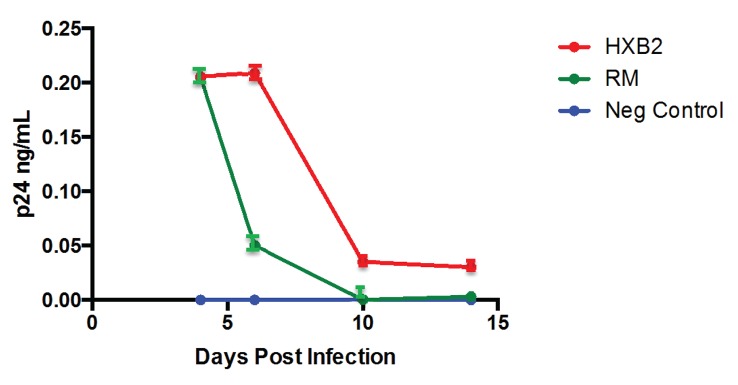
p24 is detected in the basolateral media following exposure to HIV-1. p24 antigen levels measured from the basolateral media of NHBE cells infected with HXB2 or RM virus at days 4, 6, 10 and 14 post-infection. p24 levels decreased between day 4 and day 14 for both HXB2 (from 0.296±0.002 [Mean±SD] at day 4 to 0.033 ± 0.004 at day 14) and RM (from 0.295±0.003 at day 4 to 0.000 at day 14) infected NHBE cells (n = 3).

Given evidence indicating that both CXCR4 interaction and viral fusion with the cell membrane were needed, we wanted to determine if X4 tropic virus was internalized into airway epithelium, even though they are not CD4 expressing cells. Since HIV-1 entry into epithelial cells has been previously established, the purpose of our experiment was to determine if these findings extend to the lung. NHBE cells were exposed in the basolateral media to either the X4 tropic virus HXB2 (TCID_50_ = 2.04 x10^4^) or the R5 tropic virus RM (TCID_50_ of 2.04x10^4^) and HIV levels were measured in the culture media **(Fig 5)**. For these studies we used TCID_50_ as a standardization measure instead of total viral particles as used above as we were studying internalization and potential replication of HIV. While there was a rapid decline of the antigen from day 0 levels, levels of p24 were persistently detected in the cells exposed to the X4 tropic virus compared to those exposed to an R5 tropic virus **(Fig 5)**. Following exposure to the X4 tropic virus HXB2, p24 viral antigen remained detectable in the basolateral media 14 days post infection. In contrast, by day 10 the p24 viral antigen was undetectable in the basolateral media from NHBE cells exposed to the R5 tropic virus RM. Although p24 viral antigen was detectable for up to 10 days following exposure to R5 tropic virus RM, by day 5 the levels were very low. Cells were washed and trypsinized to eliminate any adherent virus [30, 31] and assessed for viral internalization. HIV cell-associated RNA was detected in cells exposed to HXB2 but was not detected in cells exposed to RM **(Table 1)**. HIV DNA, however, was not detected in either the HXB2 exposed cells or the RM exposed cells **(Table 1)**. These data indicates that the X4-tropic virus is internalized by airway epithelial cells but without evidence of integration of viral DNA into the host genome or viral replication under our experimental conditions. R5 virus isolate, by contrast, was not even internalized in airway epithelial cells.

**Table 1:** ddPCR detection of HIV-1 HXB2 and HIV-1 RM virus DNA and Cell-associated RNA from NHBE cell cultures.

	Copies/24ul ± SE	wells detected /treated
HXB2		
Cell-associated RNA		
Day 4	50 ± 2	2/2
Day 7	0	0/2
Day 10	2	1/2
Day 14	12 ± 5	2/2
DNA		
Day 4	0	0/2
Day 7	0	0/2
Day 10	0	0/2
Day 14	0	0/2
RM		
Cell-associated RNA		
Day 4	0	0/2
Day 7	0	0/2
Day 10	0	0/2
Day 14	0	0/2
DNA		
Day 4	0	0/2
Day 7	0	0/2
Day 10	0	0/2
Day 14	0	0/2

### X4 tropic HIV creates a pro-inflammatory epithelium

**Fig 6:**
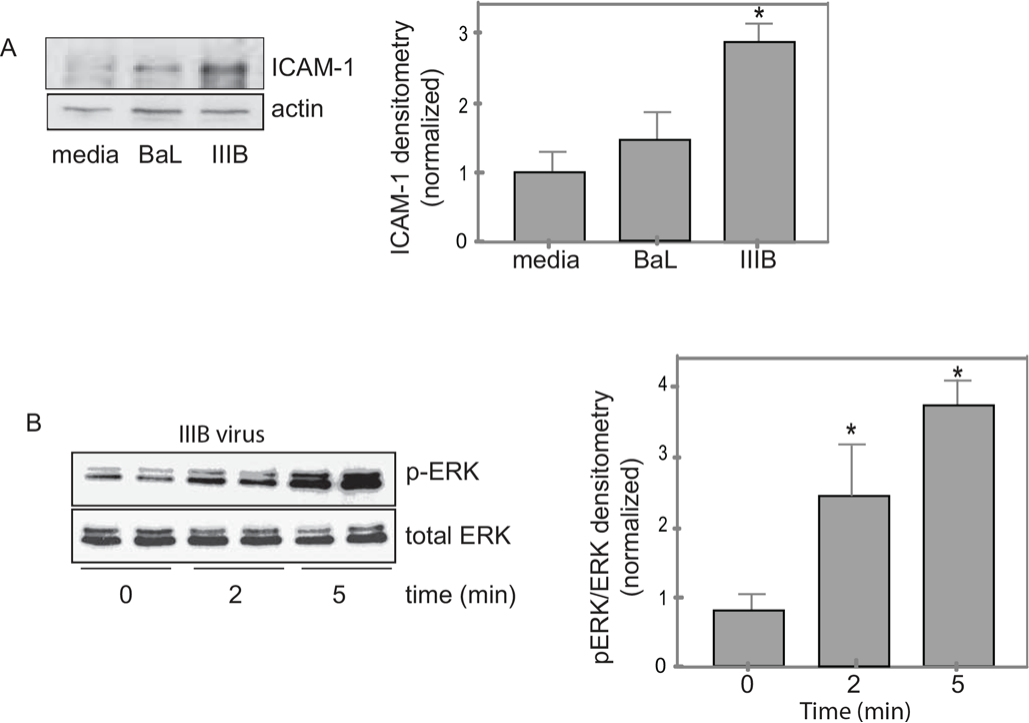
X4 tropic virus increases abundance of ICAM-1 and pERK. **A.** Following exposure to X4 tropic virus IIIB there was a statistically significant increase in the abundance if ICAM-1 that was not seen following exposure to R5 tropic virus BAL. (n = 3, *p<0.05, Anova with Bonferroni correction) **B.** After exposure to HIV-1 there was an immediate increase in the active phosphorylated ERK but no change in total ERK (n = 4, *p<0.05, Anova with Bonferroni correction).

To determine if HIV altered other aspects of the epithelium to promote the development of COPD, we assessed pro-inflammatory epithelial markers. Specifically we looked at ICAM-1 as this molecule can bind to and alter inflammatory phenotype of immune effector cells such as alveolar macrophages, a cell implicated in both HIV and COPD [32–34]. Exposure of NHBE cells to the X4 tropic virus (IIIB) but not to the R5 tropic virus (BaL) significantly increased the expression of ICAM-1 in whole cell lysates as measured by western analysis and quantified by densitometry **(Fig 6A)**. These changes were noted after four hours of exposure of p24 (5.0ng/ml) of virus. Since other epithelial pro-inflammatory cytokines are downstream of the MAPK/ERK pathway, we assessed whether HIV increased ERK activation. Exposure to the X4 tropic IIIB virus increased the expression of the active phosphorylated ERK within minutes of exposure **(Fig 6B)**, which was not found after exposure to R5 tropic BaL (not shown). There was no observed change in total ERK after exposure to HIV **(Fig 6B)**.

## Discussion

Clinically, HIV has become a more chronic condition since the advent of effective anti-retroviral therapy resulting in a tremendous increase in the long-term complications of HIV which impact patients’ health. Lung function decline and the increased susceptibility to COPD are an area of increasing concern. By studying viruses with different tropisms, we demonstrate that HIV alters the physical barrier properties of the lung epithelium potentially contributing to the increased susceptibility to lung disease in HIV patients.

**Fig 7:**
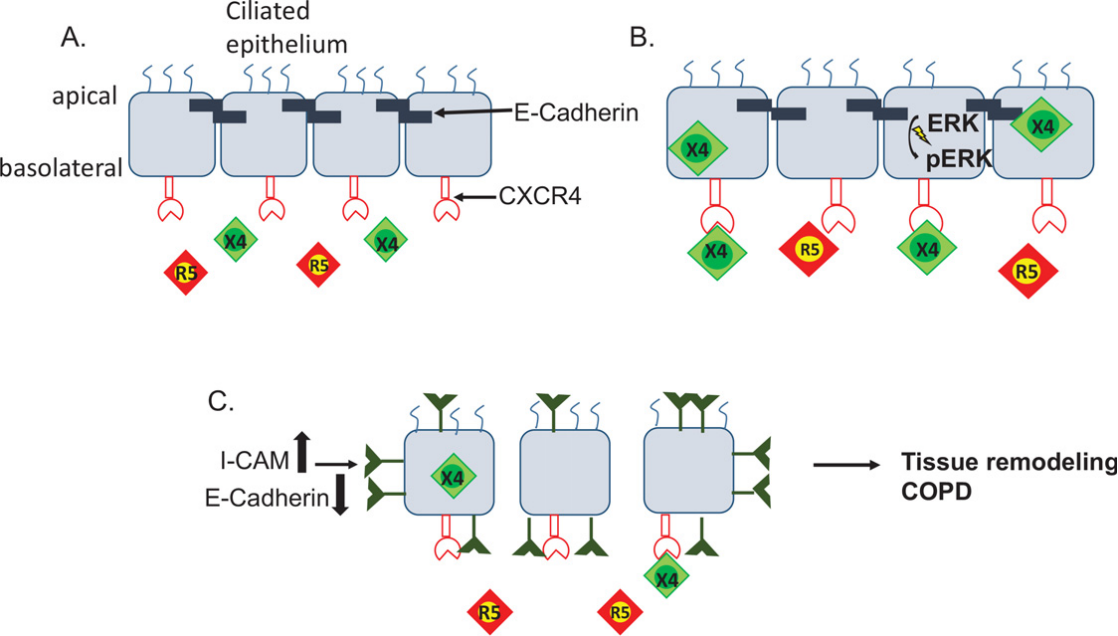
X4 tropic virus disrupts cell-cell adhesion and increases pro-inflammatory signaling leading to increased barrier permeability. **A.** The airway epithelium is formed by aggregates of ciliated cells that are joined together by adhesive proteins like E-cadherin. **B.** Our data indicates that X4 tropic viruses are able to bind to the CXCR4 receptor on the airway epithelial cells and enter these cells. Binding of X4 virus to the CXCR4 receptor leads to increase in pERK. **C.** Binding X4 tropic virus to the CXCR4 receptor decreases E-cadherin, increases monolayer permeability, and increases the expression of ICAM-1 contributing to tissue remodeling.

In this study, we used commercially available primary, differentiated lung epithelial cells and epithelial cells collected directly from patients to show that X4 tropic HIV disrupts the epithelial monolayer. While the use of primary cells limited our ability to molecularly manipulate protein targets given poor transfection efficiencies, we used highly specific, clinically utilized inhibitors to dissect out mechanisms. Our data indicates that HIV interaction with CXCR4 followed by fusion and internalization into lung epithelial cells disrupts the physical barrier and promotes the development of local inflammatory signals. This finding is significant as although most initial infections are with the R5 viruses, in advanced HIV either X4 strains or dual tropic viruses are found in the majority of patients [16, 17]. While the reason for the emergence of X4 tropic virus later in the disease course is of much debate, the ability to bind to CXCR4 allows the virus to non-productively enter a greater variety of cells and cause additional biologic impact, as we find in our study. Since COPD takes decades to develop, there is increased likelihood that patients who develop chronic lung diseases have X4 tropic virus. We propose that the increased local chronic inflammation in response to X4 tropic HIV leads to lung function decline and promotes increased susceptibility to COPD in this patient population **(Fig 7)**.

Although lung epithelial cells are not thought to express CD4, viral internalization has been demonstrated in other epithelial cells. HIV can enter other epithelial types [35–37] and compartmentalize in different tissues [36, 38]. Moreover, Popescu and colleagues showed that HIV can compartmentalize in lung mononuclear cells altering the CD4:CD8 ratio which potentially contributes to the increased susceptibility to COPD in HIV patients [39]. There is, however, limited data regarding the effects of HIV on the airway epithelium. In this study we used a quantitative measure of intracellular HIV-1 RNA, carefully controlling for cell membrane-adherent virus, to show that X4 tropic HIV not only enters the epithelium, it is also slowly released into the media for at least 14 days, thus changing the local environment and potentially acting as an ongoing source to infect immune effector cells that are recruited to the area via the pro-inflammatory signals secreted by the epithelium **(Fig 7).** The role of viral fusion and entry into the epithelial cells and the downstream consequences of X4 tropic HIV initiating the inflammatory cascade, while intriguing, are unknown at this time and will need to be further explored in future studies. The lung is thought to be a potential reservoir for HIV, and therefore it is possible that local viral presence can continue to disrupt the epithelium even during times of peripheral viral suppression [40, 41]. Moreover, by increasing epithelial pro-inflammatory signals, such as increased expression of the immune adhesion protein ICAM-1 and activation of ERK, X4 tropic HIV can have profound impact on initiating and maintaining local inflammation [42, 43] and causing tissue remodeling [44, 45], which are fundamental in the pathogenesis of COPD [46, 47]. That the X4 tropic virus causes increased expression of ICAM-1 is important given this protein’s role in recruiting other immune mediators leading to a sustained inflammatory response as well as its potential to propagate cellular infection. ICAM-1 is an adhesion molecule that mediates the transmigration of neutrophils and macrophages and has binding sites for numerous immune associated ligands. In the lungs, it has been shown to mediate the interaction between PMNs and bronchial epithelial cells [48]. HIV incorporates some of the host membrane molecules, including ICAM-1, during its budding which enhances HIV binding to cells containing LFA-1 (the counterligand to ICAM-1) as well as the viral infectivity of these cells [33, 34]. Therefore, the upregulation of ICAM-1 in response to HIV exposure not only promotes immune cell activation and inflammation which can further local damage, but also has the potential to help propagate viral infection of other cells.

Chronic HIV is associated with a high inflammatory state. Indeed, the colonic mucosa in both chronic HIV and ulcerative colitis have a similar expression pattern of the pro-inflammatory cytokines which was significantly higher than normal control mucosa suggesting that there could be a similar mechanism of inflammation in these two illnesses [49]. Nazli and colleagues suggested that the genital and intestinal epithelium respond to HIV by producing inflammatory mediators that damage the epithelial barrier. Moreover, they showed that barrier dysfunction can lead to bacterial translocation resulting in further immune activation [10]. We demonstrate that the lung epithelium also responds to HIV by increasing the production of inflammatory mediators that likely damage the barrier and lead to increased susceptibility to chronic lung disease.

At this time, we were able to perform a limited analysis of E-cadherin levels in HIV-negative and positive patients. While our samples numbers were too small for conclusive evidence, especially since patient characteristics such smoking status could not be adjusted for, there is a notable suggestion that there is a bimodal distribution of E-cadherin in HIV patients. It was particularly striking that two of the patients with higher levels of E-cadherin were being treated with the R5 inhibitor miraviroc, suggesting these patients had R5 tropic virus. This, in addition to our other data, provides rationale for a larger evaluation of viral tropism (both peripheral and in the bronchoalveolar lavage) to identify potential subpopulations of HIV patients at greater risk for lung disease.

In conclusion, this study provides evidence that HIV not only damages the airway epithelial barrier by altering cell-cell adhesion and upregulating immune modulators, but also enters the epithelial cells. These changes provide a novel mechanism for the development of chronic lung disease seen in patients with HIV.

## Materials and Methods

### Human Subject Research (involved human participants and/or tissue)

All research involving human participants have been approved by the authors' Institutional Review Board (protocols: NA_00044708 and NA_00015485), and all clinical investigation have been conducted according to the principles expressed in the Declaration of Helsinki. Written informed consent was obtained from all participants.

### Primary Airway Epithelial Cell Culture

NHBE cells (Lonza) or primary epithelial cells isolated from bronchial brushings of HIV patients were grown on 0.4μM pore inserts (Falcon) that were coated with rat tail collagen type I (Corning) at 37⁰C with 5% CO2 in bronchial epithelial growth media (Lonza). 2x10^5^cells were plated per 6-well insert and after the cells formed a confluent monolayer (approximately 1 week), the media on the apical surface was removed and the cells were allowed to differentiate on ALI for six to eight weeks before study.

### Virus Strains, infection, and drug treatment

HIV R5 tropic (ADA and BaL) and X4 tropic (IIIB and MN) strains were obtained from Dr. Blankson (JHU). HIV X4 tropic strain HXB2 was provided by the NIH AIDS Research and Reference Reagent Program, Division of AIDS, NIAID, NIH: pLAI.2, cat 2532 from Dr. Keith Peden, courtesy of the MRC AIDS Directed Program.

For drug treatment studies, cells were pretreated for 30 minutes with 1μM of either Enfuvirtide (T20), or AMD3100 (Sigma-Aldrich). In specified experiments, cells were treated with SDF-1 (Sigma). For degradation experiments, cells were pretreated with 1μM bafilomycin or 50μM chloroquine. Following pretreatment cells were exposed to HIV basolaterally. In specified experiments, HIV was inactivated with 100mM aldrithiol-2 (AT-2, Sigma-Aldrich) prior to exposure.

### Viral Entry Assay

NHBE cells were exposed to HIV as specified at 2.04x10^4^ TCID_50_. On days 4, 6, 10 and 14 post inoculation the media was removed, spun down (10min, 1320g) and the supernatant was stored at -80°C. On days 4, 7, 10 and 14 post-infection, cells were trypsinized and stored at -80°C. Cell-associated RNA and DNA were extracted from these frozen cells using AllPrep DNA/RNA Mini Kit (Qiagen, Valencia, CA) and quantified by droplet digital PCR [50]. Media p24 antigen levels were quantified using Alliance HIV ELISA Kit (PerkinElmer).

### FITC Dextran paracellular permeability assessment

Paracellular permeability was assessed using FITC Dextran assay as previously described [20]. Briefly, following treatment, basolateral media was removed from the wells and two milliliters of fresh media was added to each well. One milliliter of 4kD FitC Dextran (Sigma) at a final concentration of 10mg/ml in PBS was added to the apical surface of the cells and allowed to incubate at 37⁰C for thirty minutes. In addition, 1mL of 4kD FitC dextran was added to a cell-free insert. Following incubation, 1.5mL of basolateral media was removed from the wells and fluorescence measured in this aliquot of media using a fluorometer at excitation of 490nm and emission of 530nm. The fluorescence readings were compared to those obtained from media alone.

### RT qPCR analysis

RNA was extracted from cells using RNeasy kit (Qiagen). E-Cadherin primers were designed using BLAST (5′-TCTCTCACGCTGTGTCATCC-3′ (forward) and 5′-CACTGGATTTGTGGTGACGA-3′ (reverse)). Product was confirmed by sequencing. *GAPDH* was used as an internal control (5′-CCATCACCATCTTCCAGGAGC-3′ (forward) and 5′-CACGGAAGGCCATGCCAGTGA-3′ (reverse)). 1μg of RNA was reverse transcribed and diluted 1:100 for qPCR using SyberGreen master mix. Samples were normalized to the mRNA expression of GAPDH and results were reported either as relative expression to the housekeeping gene using the formula 2^-ΔCt^ or as fold increase using the formula 2^-ΔΔCt^

### Protein Quantification

Protein quantities were estimated using the Bradford Assay with BSA as a standard.

### Western analysis

10μg of protein was loaded onto a gel and run at 100V. Protein was transferred at 0.25A for 60minutes at 4⁰C. Following transfer, blot was blocked in 5% milk then incubated in E-Cadherin primary antibody (Cell Signaling Technology) diluted to a concentration of 1:1000. Blot was incubated in an anti-rabbit secondary antibody diluted to a concentration of 1:10,000. Bands detected using Hyglo quick spray (Denville Scientific).

### Immunofluorescence staining

Epithelial cell monolayers were fixed in 4% paraformaldehyde, permeabilized with 0.1% Triton X-100 and blocked in 20% goat serum for one hour. E-Cadherin antibody (Cell Signaling, product #**3195)**, diluted to a final concentration of 1:200 in 1%BSA, incubated on the epithelial cell monolayer for one hour at room temperature. The cells were then washed with PBS and incubated with secondary antibody (1:400, Alexa 488- goat anti-rabbit) with the addition of phalloidin- Texas Red (1:500) for one hour at room temperature. The cell monolayer was then washed and mounted on a slide. Slides were imaged using a Leica confocal microscope and images analyzed using imaging software (ImageJ, NIH).

### Statistical analysis

Values are reported as mean +/- SEM except Fig 2D, where individual values and the median for each group are shown. Multiple groups were compared using one-way ANOVA with Bonferroni correction with multiple pairwise comparisons when data was normally distributed or with Kruskal-Wallis assessment on ranks when data was not normally distributed. Two groups were compared using the student’s t-test or Mann-Whitney rank sum test when sample size or variance was not equal. Statistical analysis was performed using Sigmaplot 11.0 (Systat Software). A p<0.05 was used for significance.
